# The Upper Extremity Functional Index: Reliability and Validity in Patients with Chronic Obstructive Pulmonary Disease

**DOI:** 10.3390/ijerph182010608

**Published:** 2021-10-10

**Authors:** Ali H. Alnahdi, Ali Albarrati

**Affiliations:** Rehabilitation Sciences Department, College of Applied Medical Sciences, King Saud University, Riyadh 11433, Saudi Arabia; albarrati@ksu.edu.sa

**Keywords:** upper limb, functional limitation, psychometrics, measurement properties, COPD, activity limitation, outcome measure

## Abstract

The aim of the current study was to examine the psychometric properties of the upper extremity functional index (UEFI) in patients with chronic obstructive pulmonary disease (COPD). Seventy patients with stable COPD completed the UEFI and St. George Respiratory Questionnaire (SGRQ) and performed lung function tests in the first testing session. They completed the UEFI and the Global Rating of Change Scale in the second session, which was within ten days of the first session. The UEFI floor and ceiling effects, internal consistency, test–retest reliability, measurement error, and construct validity were examined. The UEFI was found to have no floor and ceiling effects. The UEFI was also found to have an excellent internal consistency (Cronbach’s alpha = 0.955) and an excellent test–retest reliability (ICC_2,1_ = 0.91). Totals of 4.85 points and 11.32 points represent the scale’s standard error of measurement, and a minimal detectable change at 90% confidence was used. The UEFI scores showed a significant correlation with the SGRQ activity domain (r = −0.66, *p* < 0.001) and differed significantly between participants with severe disease and those with mild disease (*p* = 0.03). The UEFI had no floor or ceiling issues, an excellent internal consistency, a good test–retest reliability, and an acceptable measurement error. The UEFI also demonstrated evidence supporting its construct validity as a measure of upper extremity-related activity limitations in patients with COPD.

## 1. Introduction

Chronic obstructive pulmonary disease (COPD) is a leading cause of disability, accounting for 2.9% of the global disability-adjusted life-years (DALYs) in all ages [1]. The impact and burden of COPD is mostly noted in individuals aged over 50 years [1]. COPD accounts for 4.7% of the global DALYs in individuals aged 50 to 74 years and 8.5% of the global DALYs in individuals of 75 years of age and older [1].

The functional hallmark of COPD is activity limitations. Patients with COPD commonly experience difficulties in performing activities of daily living (ADL) [2,3,4]. Patients with COPD have reported limitations in a wide range of activities and clearly indicated their inability to fully perform upper extremity-related activities of daily living, which consequently has a major impact on their daily life [5,6]. Patients with COPD tend to use their upper extremities less, as upper extremity activities are associated with increased metabolic and ventilatory requirements and increased symptoms [7,8]. Given their importance, limitations in upper extremity-related daily activities have been included in the International Classification of Functioning, Disability, and Health (ICF) core set for obstructive pulmonary disease [9].

The latest American Thoracic Society statement regarding pulmonary rehabilitation recommended the assessment of ADLs as part of a comprehensive patient-centered assessment of patients with COPD [10]. Janaudis-Ferreira et al. and Monjazeb et al. reviewed the patient-reported outcome measures (PROM) that have been used in the literature to quantify the ability of patients with COPD to perform ADLs [11,12]. The authors reported the use of several PROMs with no recommended single measure and also reported the limited examination of the psychometric properties of the PROMs used in patients with COPD [11,12]. None of the PROMs included in these systematic reviews specifically measured the ability of patients with COPD to perform upper extremity-related ADLs [11,12]. Therefore, the availability of PROMs with good psychometric properties that specifically measure upper extremity-related activity limitations in patients with COPD is of paramount importance.

The upper extremity functional index (UEFI) is a region-specific PROM that quantifies upper extremity-related activity limitations [13]. The UEFI has been reported to have good measurement properties in patients with upper extremity musculoskeletal disorders [14,15,16,17] and in women who have undergone breast cancer surgery [18]. The UEFI includes 20 upper extremity daily activities that are known to be relevant and important to patients with COPD [5]. To the best of our knowledge, no prior studies have examined the psychometric properties of the UEFI in patients with COPD. Therefore, the aim of the current study was to examine the psychometric properties of the UEFI in patients with COPD. We hypothesized that UEFI would demonstrate (1) no floor or ceiling effects; (2) a good to excellent internal consistency, a good test–retest reliability, and an acceptable measurement error; (3) evidence supporting its construct validity as a measure of upper extremity activity limitations in patients with COPD.

## 2. Materials and Methods

### 2.1. Setting and Participants

Participants were recruited using convenience sampling from King Fahad Medical City and King Saud University Medical City in Riyadh, Saudi Arabia. The ethical committees of both institutions provided ethical approval for the study (KSU-IRB 017E). During routine clinical visits to outpatient respiratory clinics, patients who potentially met the study’s criteria were asked to participate in the study. Participants had to fulfil the study’s inclusion criteria of being of at least 35 years of age and a confirmed COPD diagnosis (post-bronchodilator forced expiratory volume in one second to forced vital capacity ratio (FEV1/FVC) of less than 0.7) [19]. The recruited participants had experienced no exacerbation in the two weeks prior to participation. Potential participants were excluded if they were unable to read or understand Arabic language or had neurological (e.g., stroke), musculoskeletal (e.g., shoulder and knee osteoarthritis), cardiovascular (e.g., heart failure), or pulmonary disorders (other than COPD—e.g., pulmonary fibrosis) that negatively affected their functional ability. Patients who suffered from acute exacerbation of COPD or change in treatment between the testing sessions were excluded. Participants signed an informed consent form prior to participation. Data in the current study were collected (December 2019 to February 2020) by two licensed cardiopulmonary physical therapists with a cumulative clinical experience of 15 years.

### 2.2. Procedure

Participants in the current study completed the UEFI and St. George Respiratory Questionnaire and performed a lung function test in the first testing session. Participants completed the UEFI and the Global Rating of Change Scale in the second session, which was within ten days of the first session.

### 2.3. Outcome Measures

#### 2.3.1. Upper Extremity Functional Index (UEFI)

The UEFI is a patient-reported outcome measure used to quantify upper extremity-related activity limitations [13,14,20]. The UEFI has 20 upper extremity-related activities covering house-related, work-related, recreational, and sport-related activities. Patients rated their perceived difficulty in performing the 20 activities using a 0 to 4 scale, where 0 = extreme difficulty or unable to perform activity, 1 = quite a bit of difficulty, 2 = moderate difficulty, 3 = a little bit of difficulty, and 4 = no difficulty. The items’ scores were summed to result in a total score ranging from 0 to 80, with higher scores indicating better upper extremity physical function (less activity limitation). The Arabic version of the UEFI was used in the current study, and its validity and reliability have been established previously in patients with upper extremity-related musculoskeletal disorders [20].

#### 2.3.2. St. George’s Respiratory Questionnaire (SGRQ)

Health-related quality of life was measured using the SGRQ [21,22]. SGRQ items are grouped into three subscales (symptoms, activity, impacts) reflecting the three constructs measured. The scale total score and the three subscales were scored on a 0 to 100 scale, where higher scores reflect worse health conditions [21,22]. The validity and reliability of the Arabic version of the SGRQ has been reported previously in patients with COPD [23,24,25].

#### 2.3.3. Lung Function

The forced expiratory volume in one second (FEV_1_), forced vital capacity (FVC), and the FEV_1_ to FVC ratio (FEV_1_/FVC) were used as measures of lung function and were obtained using Spirometry (Vitalograph Alpha) [26]. The Global Initiative for Chronic Obstructive Lung Disease (GOLD) criteria were used to classify COPD disease severity into mild ((GOLD 1): FEV_1_% predicted ≥ 80), moderate ((GOLD 2): FEV_1_% predicted = 50–79), severe ((GOLD 3): FEV_1_% predicted = 30–49), or very severe ((GOLD 4): FEV_1_% predicted < 30) [19].

#### 2.3.4. Global Rating of Change Scale (GRC)

The GRC was used to measure the perceived magnitude of change in functional ability between the two testing sessions. A −5 (very great deal worse) to 5 (very great deal better) GRC scale was used in the current study [27]. For the purpose of the current study, GRC scores of −1 (a tiny bit worse, almost the same), 0 (no change), and 1 (a tiny bit better, almost the same) were used to indicate that there was no change in the functional ability of the participant between the two testing sessions. The test–retest reliability assessment in the current study included only those showing no change in their health status and functional ability according to the GRC.

### 2.4. Statistical Analysis

#### 2.4.1. Floor and Ceiling Effects

The percentage of participants achieving the UEFI minimum score (0) and maximum score (80) was computed. The UEFI was considered to have floor and ceiling effects if more than 15% of the participants reached the minimum and maximum scores, respectively [28].

#### 2.4.2. Internal Consistency

The internal consistency of the UEFI was examined using Cronbach’s alpha [28]. A Cronbach’s alpha value within the range of 0.70 to 0.95 would support the internal consistency of the UEFI [28,29,30].

#### 2.4.3. Test–Retest Reliability and Measurement Error

The test–retest reliability of the UEFI was examined using the Intraclass correlation coefficient model 2,1 (ICC_2,1_; two-way random effects model) [31,32]. An ICC value ≥ 0.70 would support the test–retest reliability of the UEFI [28,29,30]. The ICC is a measure of the relative reliability of the UEFI representing “the proportion of the total variance in the measurements which is because of ‘‘true’’ differences among patients” [33]. The UEFI measurement error was estimated using the standard error of measurement (SEM = error variance square root) and the minimal detectable change (MDC_90_ = 1.65 × SEM × $\sqrt{2}$) [28,31,32]. The SEM and MDC are measures of the absolute reliability of the UEFI representing “the systematic and random error of a patient’s score that is not attributed to true changes in the construct to be measured” [33]. A Bland–Altman plot with 95% limits of agreement was also used to assess the UEFI measurement error.

#### 2.4.4. Construct Validity Using Hypothesis Testing

The construct validity of the UEFI as a measure of upper extremity-related activity limitation was examined using hypothesis testing. We hypothesized that the UEFI would have moderate to strong negative correlation with the SGRQ activity domain (≤−0.4) [34]. This correlational hypothesis was examined using Spearman’s correlation coefficient. We also hypothesized that UEFI scores would differ according to the COPD disease severity. Participants with more severe disease (GOLD 3 and 4) were expected to have lower UEFI scores, indicating greater activity limitations compared to those with mild disease (GOLD 1 and 2). This hypothesis was examined using an independent *t*-test. IBM SPSS Statistics 25 (IBM Corp, Armonk, NY, USA) was used for all statistical analyses. For normally distributed and nearly normally distributed data, means and standard deviations were used as descriptive statistics, while medians and interquartile ranges were used for non-normally distributed data. Normality was checked using the visual inspection of frequency histograms and normal Q-Q plots.

#### 2.4.5. Sample Size Estimation

The recommendations of the consensus-based standards for the selection of health measurement instruments (COSMIN) was used to determine the appropriate sample size for the current study [35]. A sample size of 50 was considered by COSMIN as a good sample size for examining the measurement properties of interest in the current study (internal consistency, test–retest reliability, measurement error, and construct validity using hypothesis testing) [35].

## 3. Results

**Figure 1:**
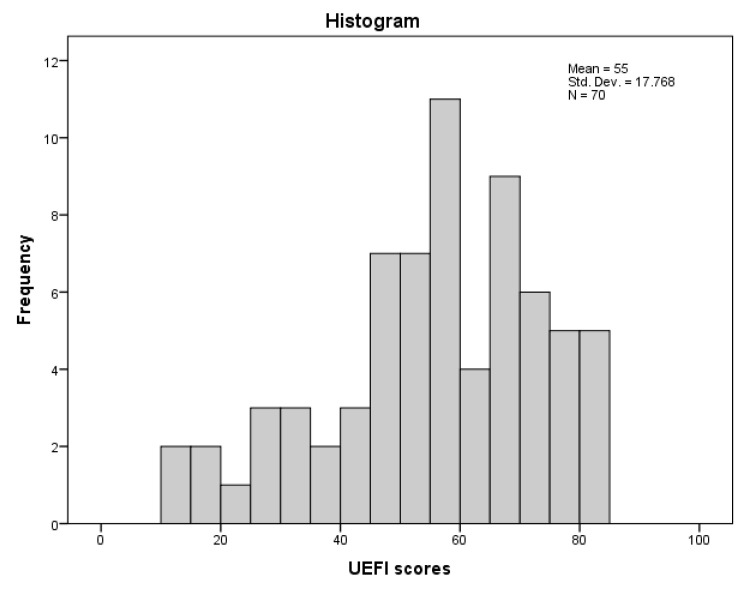
Frequency histogram of UEFI scores in the first testing session.

The characteristics of the participants in the current study (*N* = 70) are detailed in Table 1. Only one participant had missing items (2 items) in the UEFI, while the rest of the participants completed the scale with no missing items. No imputations were performed for missing items. Participants reported no issues in completing the UEFI. The distribution of the UEFI scores at the first testing session is presented in Figure 1.

### 3.1. Floor and Ceiling Effects

The UEFI had no floor effect, given that none of the participants scored 0 (minimum score). The UEFI also had no ceiling effect given that only 5 participants (7.1%) scored 80 (maximum score).

### 3.2. Internal Consistency

Cronbach’s alpha (0.955) indicated that UEFI had excellent internal consistency. The removal of one item at a time did not change the value of Cronbach’s alpha (0.951 to 0.956).

### 3.3. Test–Retest Reliability and Measurement Error

**Figure 2:**
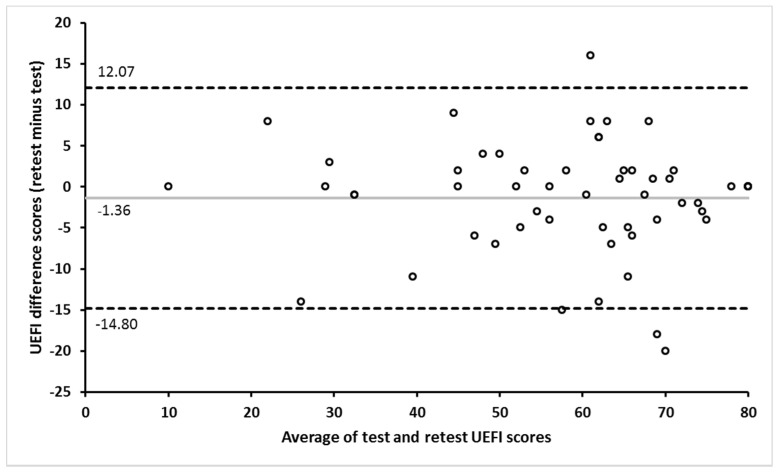
Bland–Altman plot showing the agreement between the UEFI test and retest scores. The gray horizontal line represents the mean difference between the test and retest scores. The upper and lower dashed horizontal lines represent the 95% limits of agreement.

Out of the 70 participants in the current study, 59 participants completed the UEFI in the two testing sessions, and only 4 participants out of the 59 reported a change in their functional ability between the two administrations of the UEFI. The test–retest reliability and measurement error assessment included only those showing no change (*N* = 55) in their functional ability between the two administrations of the UEFI according to their GRC scores. The time frame between the two administrations of the UEFI was 9.1±1.7 days (range: 7–13 days). The UEFI demonstrated an excellent test–retest reliability and an acceptable measurement error (Table 2). The Bland–Altman plot indicates the absence of systematic error between the two administrations of the UEFI (Figure 2).

### 3.4. Construct Validity Using Hypothesis Testing

The UEFI demonstrated significant moderate negative correlation with the SGRQ activity domain, as hypothesized (Table 3). Participants with more severe disease (GOLD 3 and 4) had lower UEFI scores (49.80 ± 19.30), indicating their greater activity limitation compared to those with mild disease (GOLD 1 and 2) (58.90 ± 15.66). The two groups differed by 9.1 points in the UEFI (95% CI; 0.76 to 17.44), with an effect size of 0.52 (*p* = 0.03).

## 4. Discussion

The current study aimed to examine the psychometric properties of the UEFI in patients with COPD. The UEFI demonstrated no floor or ceiling issues and had excellent internal consistency, good test–retest reliability, and an acceptable measurement error. The UEFI also demonstrated evidence supporting its construct validity as a measure of upper extremity-related activity limitations in patients with COPD.

Patients with COPD usually have limited upper extremity function due to the increased breathlessness encountered during upper extremity activities and increased upper extremity muscles fatigue [36]. Through the use of upper extremity specific PROM in patients with COPD, capturing upper extremity-related activity limitation is imperative given the reported upper extremity activity limitations and the relevance of these limitations to the daily functioning of this patient population [5,6]. The assessment of upper extremity function and designing a structured exercise training are recommended components of the rehabilitation of patients with COPD [10]. These types of exercise training essentially aim to improve upper extremity physical functioning, which ideally should be reflected in the ability of patients to perform upper extremity-related ADLs. An upper extremity region-specific outcome measure, such as the UEFI, would help health care providers to quantify and monitor changes in the ability of patients with COPD to perform upper extremity-related ADLs. Although it was initially developed and used in patients with upper extremity musculoskeletal disorders [13], the UEFI measures an important construct (upper extremity-related activity limitations) relevant to patients with COPD. The UEFI items cover ADLs that are reported by patients with COPD to be important and relevant to their daily functioning [5]. As part of pulmonary rehabilitation, the UEFI can be used to measure the baseline level of upper extremity functional ability and monitor the change in upper extremity functional ability as a result of the rehabilitation program.

Consistent with our hypothesis, the UEFI exhibited an excellent internal consistency. The value of Cronbach’s alpha (0.955) was at the upper end of the recommended values, suggesting a possible redundancy between items [28,30]. However, the removal of any single item at a time did not change the scale’s internal consistency; thus, no item was removed from the scale. In line with our findings, the UEFI has been reported to have excellent internal consistency in patients with upper extremity musculoskeletal disorders (Cronbach’s alpha ranging from 0.89 to 0.96) [13,15,17,20].

The measurement error of the UEFI in patients with COPD was quantified in the current study using SEM and MDC_90_. The SEM reported in the current study (4.85) represents 6.1% of the UEFI score range, while the reported MDC_90_ (11.32) represents 14.2% of the UEFI score range. These values indicate that measurement error of the UEFI represents only a small portion of the total scale range, thereby supporting the acceptability of this magnitude of measurement error. With the repeated administration of the UEFI in patients with COPD, a change of at least 11.32 points in the UEFI is required to be considered as a true change in a patient’s upper extremity activity limitation. The SEM of the UEFI in patients with upper extremity-related musculoskeletal disorders has been reported to range from 4.1 to 5.5 points, while the MDC_90_ has been reported to range from 9.4 to 17.6 points [13,14,20,37]. In women who have undergone breast cancer surgery, the UEFI was reported to have an SEM of 4.8 points and an MDC_90_ of 11.1 points [18]. The magnitude of measurement error reported in these studies is similar to the magnitude of the measurement error reported in the current study.

The UEFI is presumed to measure one construct—that is, upper extremity activity limitation. Based on this assumption, we examined the construct validity of the UEFI by examining predefined hypotheses regarding the expected correlation of the UEFI with the SGRQ activity domain as well as the expected differences in the UEFI scores according to COPD severity [35]. The results of the current study were consistent with our predefined hypotheses, providing evidence to support the construct validity of the UEFI as a measure of upper extremity activity limitation in patients with COPD.

The UEFI was hypothesized to have a moderate to strong negative correlation with the SGRQ activity domain. This hypothesized correlation was defined given that both outcomes assess a similar construct—that is, activity limitation (physical function). Higher scores in the UEFI indicate less severe activity limitation (better physical function), while higher scores in the SGRQ activity domain indicate a higher level of activity limitation (worse physical function); thus, the expected direction of correlation was negative. A significant correlation was observed between the two measures; this was consistent with our predefined hypothesis in terms of the strength of the correlation and its direction. To the best of our knowledge, no prior studies have reported a correlation between the UEFI and the SGRQ activity domain. On the other hand, the UEFI has been found to correlate with other measures of activity limitation in patients with upper extremity musculoskeletal disorders [13,14,15,17,20,37,38,39] and in women who have undergone breast cancer surgery [18]. The pattern of correlation of the UEFI with the other measures of activity limitation in these studies is similar to the pattern of correlation between the UEFI and the SGRQ activity domain observed in the current study in patients with COPD.

In the current study, the UEFI scores differed according to COPD severity, as hypothesized a priori. Patients with more severe COPD (GOLD 3 and 4) had lower UEFI scores, indicating worse upper extremity self-reported physical function compared to those with mild COPD (GOLD 1 and 2). The magnitude of the difference between both groups was clinically meaningful, as suggested by the moderate effect size. A number of previous studies have reported findings regarding disease severity and self-reported physical function that are consistent with the findings reported in the current study [3,40,41]. The progression of COPD severity usually impacts a patient’s function and symptoms; subsequently. patients tend to reduce their participation in activities associated with upper extremity due to their link with the increased work of breathing [7,8,36].

This study has a number of limitations that should be acknowledged. The majority of participants in the current study had either moderate (GOLD 2) or severe COPD disease (GOLD 3); thus, the results of the current study should be interpreted with caution for patients with mild (GOLD 1) and severe disease (GOLD 4), given that they represented a small proportion of the participants. The current study did not examine the responsiveness of the UEFI in patients with COPD. Based on this, future studies are needed to examine whether the UEFI is able to detect changes in upper extremity activity limitation over time in patients with COPD.

## 5. Conclusions

The UEFI demonstrated no floor or ceiling issues and had an excellent internal consistency, a good test–retest reliability, and an acceptable measurement error. The UEFI also demonstrated evidence supporting its construct validity as a measure of upper extremity-related activity limitations in patients with COPD. Owing to its simplicity and good psychometric properties, the UEFI can be utilized to quantify upper extremity activity limitations in patients with COPD in routine clinical practice.

## Figures and Tables

**Table 1:** Characteristics of participants (*N*= 70).

Variable	Mean ± SD or Median (IQR; Q1, Q3) or *N* (%)
Age (year)	63.23 ± 11.10
Sex	
Male	56 (80)
Female	14 (20)
Height (m)	1.65 ± 0.08
Mass (Kg)	78.88 ± 15.26
Body mass index (Kg/m^2^)	29.05 ± 5.35
Smoking Status	
Current smoker	17 (24.3)
Previous smoker	39 (55.7)
Never smoked	14 (20)
Lung function	
FEV_1_ (L)	1.57 ± 0.55
FVC (L)	2.81 (1.28; 2.22, 3.49)
FEV_1_/FVC ratio	56.35 (21.45; 44.55, 66.0)
FEV_1_% predicted	55.99 ± 14.98
COPD severity *	
Mild (GOLD 1)	4 (5.7)
Moderate (GOLD 2)	36 (51.4)
Severe (GOLD 3)	27 (38.6)
Very severe (GOLD 4)	3 (4.3)
UEFI (0–80)	55.0 ± 17.77
UEFI in patients with mild to moderate disease (GOLD 1 and 2)	58.90 ± 15.66
UEFI in patients with severe to very severe disease (GOLD 3 and 4)	49.80 ± 19.30
SGRQ	
Symptoms (0–100)	53.55 ± 23.46
Activity (0–100)	59.46 (38.93; 34.11, 73.04)
Impact (0–100)	45.88 (47.25; 17.99, 65.25)
Total (0–100)	49.14 ± 24.51

IQR = interquartile range, Q1 = first quartile, Q3 = third quartile, FEV_1_ = forced expiratory volume in one second, FVC = forced vital capacity, COPD = chronic obstructive pulmonary disease. * Classified according to the Global Initiative for Chronic Obstructive Lung Disease (GOLD). UEFI = upper extremity functional index. SGRQ = St. George’s Respiratory Questionnaire.

**Table 2:** Test–retest reliability and measurement error (*N* = 55).

Outcome Measure	Test Mean ± SD	Retest Mean ± SD	Mean Difference * (95% CI)	ICC_2,1_ (95% CI)	SEM	MDC_90_
UEFI	58.62 ± 16.52	57.25 ±16.12	1.36 (−0.49 to 3.22)	0.91 (0.85 to 0.95)	4.85	11.32

* Test score minus the retest score. UEFI = upper extremity functional index, ICC = intraclass correlation coefficient (two-way random model for agreement), SEM = standard error of measurement for agreement, MDC_90_ = minimal detectable change with 90% confidence.

**Table 3:** Correlation between UEFI and other measures (*N* = 70).

Variable	*rs* (95% CI)	*p*
SGRQ Symptoms	−0.35 (−0.11 to −0.58)	0.003
SGRQ Activity	−0.66 (−0.46 to −0.80)	<0.001
SGRQ Impact	−0.63 (−0.45 to −0.76)	<0.001
SGRQ Total	−0.63 (−0.45 to −0.77)	<0.001

*rs* = Spearman’s correlation coefficient; CI = confidence interval; UEFI = upper extremity functional index, SGRQ = St. George’s Respiratory Questionnaire.

## Data Availability

The data presented in this study are available from the corresponding author on reasonable request.
